# Gene 33/Mig6 inhibits hexavalent chromium-induced DNA damage and cell transformation in human lung epithelial cells

**DOI:** 10.18632/oncotarget.6866

**Published:** 2016-01-09

**Authors:** Soyoung Park, Cen Li, Hong Zhao, Zbigniew Darzynkiewicz, Dazhong Xu

**Affiliations:** ^1^ Department of Pathology, School of Medicine, New York Medical College, Valhalla, NY, USA; ^2^ Brander Cancer Research Institute, School of Medicine, New York Medical College, Valhalla, NY, USA

**Keywords:** Gene 33/Mig6, chromium, DNA damage, carcinogenesis, lung

## Abstract

Hexavalent Chromium [Cr(VI)] compounds are human lung carcinogens and environmental/occupational hazards. The molecular mechanisms of Cr(VI) carcinogenesis appear to be complex and are poorly defined. In this study, we investigated the potential role of Gene 33 (ERRFI1, Mig6), a multifunctional adaptor protein, in Cr(VI)-mediated lung carcinogenesis. We show that the level of Gene 33 protein is suppressed by both acute and chronic Cr(VI) treatments in a dose- and time-dependent fashion in BEAS-2B lung epithelial cells. The inhibition also occurs in A549 lung bronchial carcinoma cells. Cr(VI) suppresses Gene 33 expression mainly through post-transcriptional mechanisms, although the mRNA level of gene 33 also tends to be lower upon Cr(VI) treatments. Cr(VI)-induced DNA damage appears primarily in the S phases of the cell cycle despite the high basal DNA damage signals at the G2M phase. Knockdown of Gene 33 with siRNA significantly elevates Cr(VI)-induced DNA damage in both BEAS-2B and A549 cells. Depletion of Gene 33 also promotes Cr(VI)-induced micronucleus (MN) formation and cell transformation in BEAS-2B cells. Our results reveal a novel function of Gene 33 in Cr(VI)-induced DNA damage and lung epithelial cell transformation. We propose that in addition to its role in the canonical EGFR signaling pathway and other signaling pathways, Gene 33 may also inhibit Cr(VI)-induced lung carcinogenesis by reducing DNA damage triggered by Cr(VI).

## INTRODUCTION

Cr(VI) compounds are well documented human lung carcinogens [1-3]. Occupational exposure, mainly through inhalation, during industrial processes such as chrome plating, stainless steel production, and chrome pigment manufacturing are the main sources of human contact with Cr(VI) [1-3]. Cr(VI) is a genotoxic agent that induces DNA damage [2, 4-8]. The toxic effect of Cr(VI) is believed to be a result of oxidative stresses generated during its reduction process [7]. Exposure of cells to Cr(VI) provokes a typical DNA damage response that leads to cell cycle arrest, apoptosis, and senescence [4-6, 8-10]. Interestingly, despite the ability of forming potentially mutagenic Cr(III)-DNA ternary adducts during its intracellular reduction process [9], Cr(VI) appears to be a weak mutagen [5]. Consistently, lung cancers associated with Cr(VI) exposure exhibit few mutations of the key oncogenes or tumor suppressors [2]. The genotoxic effect of Cr(VI) is more likely represented by genomic instability in forms of chromosome instability and/or microsatellite instability [5]. Epigenetic effects of Cr(VI) which lead to altered histone methylation, histone acetylation, and DNA methylation have also been reported and believed to play significant roles in lung carcinogenesis [11, 12]. Despite these previous efforts, the specific mechanisms, particularly the specific intracellular molecular mediators of Cr(VI) carcinogenesis, remain poorly defined.

DNA damage triggers phosphorylation of histone H2AX at Ser139 (γH2AX) by The ATM family protein kinases as part of the DNA damage response [13-15]. Generation of γH2AX is considered a hallmark of DNA double strand breaks (DSBs), the most detrimental form of DNA damage [16, 17]. The DNA damage response serves to initiate DNA repair or, in case of irreparable DNA damage, to eliminate cells carrying the damage through apoptosis or senescence [13-15]. Improper DNA repair can lead to genomic instability, characterized by DNA mutations and/or chromosome instability [18]. It is conceivable that cellular components that affect the generation or repair of DNA damage can have important impact on genomic stability and carcinogenesis.

Gene 33 is an inducible adaptor protein containing multiple domains for protein-protein interaction and signal transduction [19, 20]. The most significant function of Gene 33 characterized thus far is to bind to the kinase domain of ErbB receptor tyrosine kinases thereby inhibiting their kinase activities and the downstream signaling pathways [20-22]. Gene 33 can also induce apoptosis by activating the protein tyrosine kinase cAbl and inhibiting the ErbB-PI3K-AKT survival pathway [23, 24]. Gene 33 may contribute to replicative and oncogene-induced cell senescence through its inhibition of the EGFR signaling [25]. Gene 33 has also been shown to regulate cell migration through the HGF/c-Met signaling pathway [26]. Thus, Gene 33 regulates multiple signaling pathways that are important for tumorigenesis and tumor progression [27].

Increasing evidence supports a role of Gene 33 as a bona fide tumor suppressor in the lung. Nonsense or missense mutations of *gene 33 (errfi1)* have been detected in lung cancers [28]. Reduced or loss of Gene 33 expression has been reported in significant number of human lung cancer samples and cell lines [28, 29]. *Gene 33* null mice tend to develop spontaneous lung adenoma and adenocarcinoma [28, 30]. *Gene 33* is located at chromosome 1p36.32 where loss of heterozygosity occurs frequently in lung cancer and associated with tobacco smoking [31].

Despite these findings, an assessment of its role in lung carcinogenesis in response to a relevant environmental lung carcinogen has not been conducted. Here we report that Gene 33 protein expression can be significantly suppressed by Cr(VI) in both lung epithelial (BEAS-2B) and lung cancer (A549) cells through both transcriptional and post-transcriptional mechanisms. Cr(VI) induces a DNA damage response, which occurs mainly in the S phase of the cell cycle. Knockdown of Gene 33 by siRNA elevates the Cr(VI)-induced DNA damage in BEAS-2B cells, which led to elevated micronucleus formation and cell transformation. Our data reveal a novel function of Gene 33 in regulating Cr(VI)-induced DNA damage and a potential involvement of this protein in Cr(VI)-mediated genotoxicity and carcinogenesis.

## RESULTS

### Cr(VI) suppresses Gene 33 expression

**Figure 1:**
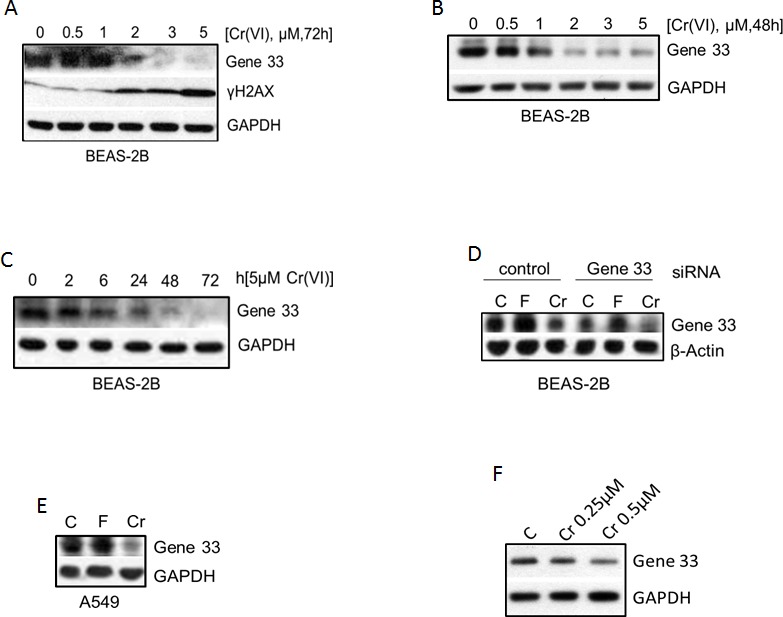
Cr(VI) suppresses the protein level of Gene 33 **A.** BEAS-2B cells were treated with the indicated concentrations of Cr(VI) for 72 hours and harvested for total cellular proteins with 1x sample buffer. Total cellular proteins were subjected to Western blot as described in Materials and Methods with antibodies toward the indicated proteins. GAPDH or β-actin was used a loading control. **B.** Same as A with 48h instead. **C.** BEAS-2B cells were treated with 5 μM Cr(VI) for indicated time periods and then processed as in A. **D.** BEAS-2B cells were transfected with either a scrambled siRNA (control) or an siRNA targeting Gene 33 as described in Materials and Methods. Forty eight hour after transfection, cells were treated with 20% FBS (F) or Cr(VI) for additional 48 hours and processed as in A. **E.** A549 cells were left untreated or treated with 20% FBS or 5 μM Cr(VI) for 48 hours. Cells were then processed as in A. **F.** BEAS-2B cells were cultured for 2 months (passed every three days) using media containing 0, 0.25, or 0.5 μM Cr(VI). Cells were harvested and processed at in A.

As reduced expression of tumor suppressor proteins is often associated with tumorigenesis, we checked whether the expression of tumor suppressor protein Gene 33 is regulated by Cr(VI). We treated BEAS-2B cells with different concentrations of Cr(VI)(we used Na_2_CrO_4_ throughout the study) for different periods of time. We observed that Cr(VI) suppressed the protein level of Gene 33 in a dose- and time-dependent fashion, with significant inhibition started at 1μM and 24 hours, respectively (Figure 1A, 1B, 1C). A dose-dependent increase of γH2AX was also observed with significant elevation at 2μM (Figure 1A). The activation of γH2AX indicates that Cr(VI) induces DNA damage in the form of DNA double strand breaks (DSBs), confirming the previously published observations [4, 6, 10, 32]. Figure 1D shows that Cr(VI) could further inhibit Gene 33 expression after Gene 33 knockdown by RNAi. Cr(VI) also inhibited Gene 33 expression in A549 cells (Figure 1E). In both BEAS-2B and A549 cells Gene 33 could be induced by bringing FBS content to 20% in normal DMEM (Figure 1D & 1E), consistent with the finding that Gene 33 is a mitogen inducible protein [20, 24]. We further examined whether long term exposure to low concentrations of Cr(VI) also affects the level of Gene 33 protein. We treated BEAS-2B cells with 0.25 or 0.5 μM Cr(VI), two sub-lethal doses of Cr(VI) to BEAS-2B cells [33], for 2 months followed by checking the levels of Gene 33 protein. As shown in Figure 1F, Cr(VI) treatments led to a dose-dependent reduction of Gene 33 protein levels in these cells. Collectively, our data demonstrate that the Gene 33 protein level is suppressible by Cr(VI) in lung epithelial and lung cancer cells.

### Gene 33 expression is regulated at multiple levels by Cr(VI)

**Figure 2:**
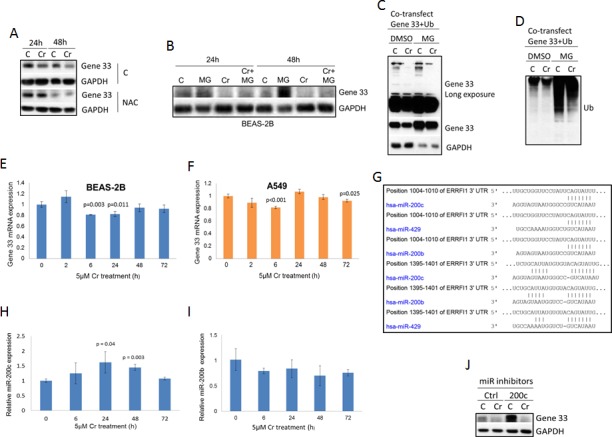
Gene 33 expression is regulated by Cr(VI) through multiple mechanisms **A.** BEAS-2B cells were either left untreated or treated with 5 μM Cr(VI), with or without presence of 20mM NAC for 24 or 48 hours. Cells were then harvested and Western blotted with antibodies toward the indicated proteins. **B.** BEAS-2B cells where left untreated or treated with 10μM MG132, 5 μM Cr(VI), or 10μM MG132 plus 5 μM Cr(VI) for 24 or 48 hours as indicated (MG132 was added 6 hours before the end of the treatment). Cells were then processed as in A. **C.** BEAS-2B cells were co-transfected with plasmids containing FLAG-tagged Gene 33 and FLAG-tagged ubiquitin. Twenty fours later, cells were left untreated or treated with 5 μM Cr(VI), with or without presence of 10 μM MG132. Cells were then processed as in A. **D.** Total cellular proteins from C were Western blotted with an antibody toward FLAG. J. BEAS-2B cells were transfected with an miRNA mimics (Ctrl) or an miR200c inhibitor (200c). Seventy hours after transfection, the cells were treated with or without Cr(VI) for 48hours. The Gene33 expression levels were then determined by Western blot. Data are presented as mean +/− SD (E,F,H,I).

We next studied how Cr(VI) regulates Gene 33 expression. It is well documented that Cr(VI) generates the oxidative stress, which is likely the root cause of its toxicity [7]. We therefore checked whether suppression of the oxidative stress affects the level of Gene 33 protein. We find that N-acetylcysteine (NAC), a free radical scavenger, prevented the Cr(VI)-induced inhibition of the Gene 33 protein level at both 24 and 48 hours after Cr (VI) and NAC co-treatments. (Figure 2A). This result indicates that the inhibition of the Gene 33 protein level was caused by Cr(VI)-mediated generation of oxidative stresses rather than by direct action of Cr(VI). Of note, NAC alone was able to strongly inhibit Gene 33 expression (Figure 2A, NAC group). This would indicate that constitutive level of expression of this gene, in untreated cells, is facilitated by endogenous oxidants.

Gene 33 is an inducible protein whose expression is tightly controlled by mitogenic and stress signals [19, 20]. In addition, Gene 33 contains two PEST domains suggesting that the ubiquitin/proteasome-mediated protein degradation mechanism may be involved in regulating the Gene 33 level [19, 20]. To explore this, we used MG132, an inhibitor of the 26S proteasome [34], to examine whether the proteasome-mediated protein degradation pathway plays a role in the observed reduction of Gene 33 protein level upon Cr(VI) treatments. As expected, MG132 was able to significantly raise the basal Gene 33 protein level in BEAS-2B cells (Figure 2B). However, MG132 only modestly reversed the effect of Cr(VI) on Gene 33 expression (Figure 2B). These data indicate that while Gene 33 is indeed subject to regulation by proteasome-mediated pathway, the inhibition of Gene 33 expression by Cr(VI) can only be partially attributed to this mechanism. To further test this notion, we ectopically co-overexpressed FLAG-tagged Gene 33 with ubiquitin in BEAS-2B cells to see whether the ectopically expressed Gene 33 can be ubiquitinated by ectopically expressed ubiquitin. Indeed, as shown in Figure 2C, Gene 33 was ubiquitinated by ectopically expressed ubiquitin, as indicated by higher molecular weight proteins detected by anti-Gene 33 antibody. Inhibition of the 26S proteasome by MG132 elevated the level of Gene 33 ubiquitination (Figure 2C). Consistently, the Gene 33 protein level was strongly elevated by MG132. These data confirm that Gene 33 is indeed subjected to regulation by ubiquitin-proteasome system. However, albeit more modest compared to the endogenous Gene 33, Cr(VI) could still suppress the level of ectopically expressed Gene 33 protein with or without the presence of MG132, confirming that the proteasome-mediated pathway may only partially responsible for the suppression. Moreover, Cr(VI) strongly reduced the level of ubiquitinated Gene 33 and inhibition of the 26S proteasome by MG132 did not significantly reverse this reduction (Figure 2C). These data are against the notion that Cr(VI) destabilizes the Gene 33 protein by enhancing its ubiquitination. Instead, they appears to suggest that Cr(VI) either activates a mechanism independent of the 26S proteasome system to degrade the ubiquitinated Gene 33 or Cr(VI) serves as a activator of the 26S proteasome that can partially overcomes the inhibitory effect of MG132, or both.

As expected, ectopic expression of ubiquitin led to strong global ubiquitination of cellular proteins, showing as strong ubiquitin signals detected by the FLAG antibody (Figure 2D). Inhibition of the 26S proteasome by MG132 strongly enhanced the global level of ubiquitination (Figure 2D). Of note, Cr(VI) appears to reduce the global ubiquitination with and without presence of MG132 (Figure 2D). These data are consistent with above prediction that Cr(VI) activates an alternative mechanism or the 26S proteasome itself in a MG132-independent fashion that degrades at least some ubiquitinated proteins.

It appears that endogenous Gene 33 was more strongly inhibited by Cr(VI) than the ectopically expressed one (compare Figure 1 with Figure 2C). This suggests that transcriptional or other post-transcriptional mechanisms may also be involved in the regulation of the expression of the endogenous Gene 33. We therefore accessed whether Gene 33 is regulated by Cr(VI) at the mRNA level. Using real-Time PCR, we observed that Cr(VI) has a tendency to modestly inhibit the Gene 33 mRNA level in both BEAS-2B and A549 cells, although significant inhibition only occurred at certain time points after treatments (Figure 2E and 2F).

Given the relatively weak effect of Cr(VI) on Gene 33 mRNA, we searched for other potential mechanisms. Gene 33 has been shown to be regulated by microRNAs, specifically miR-200 and miR-148 [35, 36]. We used TargetScan to search for potential microRNA targets on the 3′UTR of the Gene mRNA. As shown in Figure 2G, multiple potential targets for miR-200 family microRNAs present at the 3′UTR of Gene 33 mRNA, including those for miR-200b, miR-200c, and miR-429. We then measured the expression of miR-200b and miR-200c in BEAS-2B cells in response to Cr(VI) using real-Time PCR. We find that miR-200c but not miR-200b was modestly induced by Cr(VI) (Figure 2H and 2I), suggesting that miR-200c may contribute to suppression of Gene 33 by Cr(VI). To confirm this observation, we transfected a miR-200c inhibitor (Life Technologies) to see whether inhibition of miR-200c would overcome the suppression of Gene 33 by Cr(VI). Surprisingly, while miR-200c strongly elevated the level of Gene 33 protein, it failed to overcome the inhibition by Cr(VI). These data indicate that the basal level of Gene 33 is indeed regulated by miR-200c but its suppression by Cr(VI) is not mediated by the modest induction of miR-200c in response to Cr(VI). Taken together, our data indicate that in lung epithelial and lung cancer cells Gene 33 expression are regulated by Cr(VI) through a combination of mRNA expression, protein degradation, and possibly other unidentified translational/post translational mechanisms.

### Cr(VI) induces DNA damage responses

**Figure 3:**
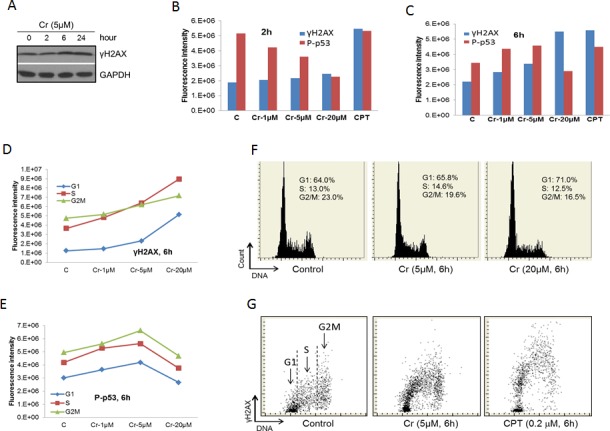
Cr(VI) induces the DNA damage response **A.** BEAS-2B cells were treated with 5 μM Cr(VI) for the indicated time periods. At the end of the treatments, total proteins were extracted from the cells and subjected to Western blot with antibodies against the indicated proteins. **B**. & **C**. BEAS-2B cells on chamber slides were treated with Cr(VI) at the indicated concentrations for 2 hours **B.** or 6 hours **C.** followed by LSC as described in Materials and Methods. The total levels of γH2AX and P-p53 were plotted against the concentration of Cr(VI). **D**. & **E**. Data from B and C were analyzed by splitting the cells in different phases of the cell cycle according to the DNA content. The levels of γH2AX **D.** or P-p53 **E.** in cells of different phases of the cell cycle were plotted against the concentration of Cr(VI). **F**. Examples of the readout of the cell cycle analysis showing percentages of cell in different phases of the cell cycle. **G**. Examples of the readout of the γH2AX measurement.

Cr(VI) has been shown to induce DNA damage [2-8]. To confirm this, we treated BEAS-2B cells with Cr(VI) and checked the level of γH2AX, a hallmark of DSBs [17], using Western blot. We find that the level of γH2AX was significant elevated upon Cr(VI) treatment for 6 and 24 hours (Figure 3A). In contrast, 2 hour Cr(VI) treatment generate minimal amount of γH2AX. This time course is significantly slower than a typical chemotherapeutic drug that interferes with the DNA replication process, which generates a peak γH2AX response at within 2 hours [17, 37]. These results are consistent with the notion that Cr(VI) induces DNA damage indirectly through generation of oxidative stresses [7].

We next used Laser Scanning Cytometry (LSC) to further examine the effect of Cr(VI) on the DNA damage response in BEAS-2B cells. We observed that Cr(VI) had relatively weak effect on the γH2AX level at all Cr(VI) concentrations at 2 hour treatment while produced strong response at 6 hour (Figure 3B and 3C). In contrast, Camptothecin, a chemotherapeutic drug that inhibits Topoisomerase I (used as a positive control to detect H2AX phosphorylation) [37, 38], strongly increased the γH2AX level at both 2 and 6 hours (Figure 3B and 3C). These data confirm our results using Western blot (Figure 3A). We also simultaneously measured the phosphorylation level of p53 at serine 15 (P-p53) in these experiments. Interestingly, there was a dose-dependent reduction of P-p53 for the 2 hour treatment compared to a general increase for the 6 hour treatment, except at the 20 μM concentration (Figure 3B and 3C). The differential behavior between γH2AX and P-p53 may reflect the fact that serine 15 of p53 is a target of a more diverse spectrum of kinases while H2AX is mainly phosphorylated by the ATM family kinases that are directly associated with DNA damage [16].

Further analysis of the data on the 6 hour treatment by separating different phases of the cell cycle reveals that γH2AX mainly presented at the S and G2/M phases of the cell cycle (Figure 3D). However, the dose-dependent increases in γH2AX were much more pronounced for cells in G1 and S phases than those in G2M phase (Figure 3D), indicating that Cr(VI)-induced DSBs mainly occur in the S and apparent the G1 phases of the cell cycle. However, we believe that the apparent strong induction of DSBs in cells of G1 phase actually caused by cells entering S phase (see Discussion). In contrast to γH2AX, the dose-dependent changes in P-p53 were similar among different phases (Figure 3E). Cr(VI) treatment also led to cell cycle arrest at the G1 phase of the cell cycle in a dose-dependent fashion (Figure 3F). Figure 3G shows sample readouts of LSC.

### Gene 33 knockdown promotes Cr(VI)-induced DNA damage

**Figure 4:**
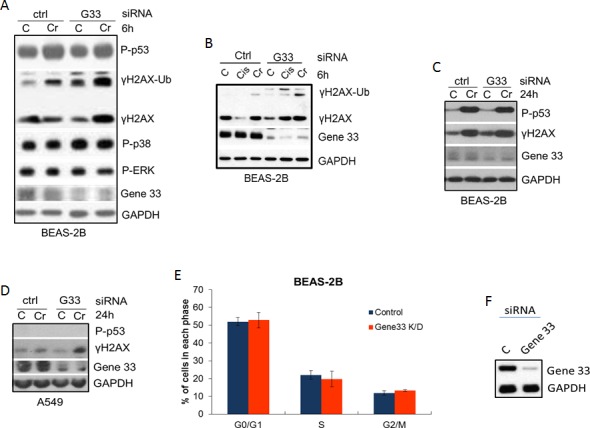
Gene 33 depletion elevates Cr(VI)-induced DNA damage but does not affect cell cycle progression **A.-C**. BEAS-2B **A.** & **B.** or A549 **C.** cells were transfected with scrambled (Ctrl) siRNA oligo or an siRNA oligo for Gene 33 (G33). Forty eight hours after transfection, cells were either left untreated or treated with 5 μM Cr(VI) for 6 hours (A&B) or 24 hours **C.** & **D.**. Cells were harvested for total proteins at the end of the treatments. Total protein lysates were subjected to Western blot with antibodies against the indicated proteins. **D**. & **E**. BEAS-2B cells transfected with either control or Gene 33 siRNA oligos as in A, B&C. Cells were then passed 48 hours after transfection. Twenty four hours later, Cells were stained with DAPI followed by cell cycle analysis. Data are presented as mean +/− SD. An aliquot of cells from each transfection was collected for Western blot to check the protein level of Gene 33 (F).

Given that Cr(VI) suppresses Gene 33 expression and induces DNA damage, we checked whether Gene 33 plays a role in Cr(VI)-induced DNA damage. We transiently knockdown Gene 33 using siRNA and measured its effect on the Cr(VI)-induced DNA damage response. We find that knockdown of Gene 33 in BEAS-2B cells elevated the level of Cr(VI)-induced DNA damage, as indicated by increased γH2AX signals in cells transfected with an siRNA oligo for Gene 33 compared to those transfected with a scrambled oligo (Figure 4A, 4B, 4C, 4D). This effect occurred after both 6 hour and 24 hour Cr(VI) treatments (Figure 4A, 4B, 4C). In contrast, while Cr(VI) strongly induced P-p53, Gene 33 knockdown had limited effect on this phosphorylation (Figure 4A and 4C). We also checked the activities of p38 and ERK by measuring their phosphorylation levels as these kinases have been implicated in the phosphorylation of p53 in response to Cr(VI) in A549 cells [39]. We find that while Gene 33 knockdown slightly increased the level of phospho-p38, the Cr(VI) treatments had limited effect on it (Figure 4A). The level of phosphorylated ERK appeared the same among different treatments (Figure 4A). A similar effect of Gene 33 on γH2AX was also observed in A549 cells, where Gene 33 knockdown also enhanced the level of γH2AX in response to Cr(VI) treatment (Figure 4D). Of note, γH2AX signals in A549 cells appeared to be weaker at both the basal level and in response to Cr(VI) treatments (Figure 4D). In addition, we could hardly detect the P-p53 signal in A549 cells with or without Cr(VI) treatment (Figure 4D). These data suggest that A549 may be more resistant to Cr(VI)-induced DNA damage than BEAS-2B cells. We also find that Gene 33 knockdown alone had limited effect on cell cycle progression of BEAS-2B cells under normal growth condition (Figure 4E and 4F). Collectively, our data indicate that Gene 33 inhibits Cr(VI)-induced DNA damage in lung epithelial and lung cancer cells.

### Gene 33 ablation enhances Cr(VI)-induced micronucleus formation

**Figure 5:**
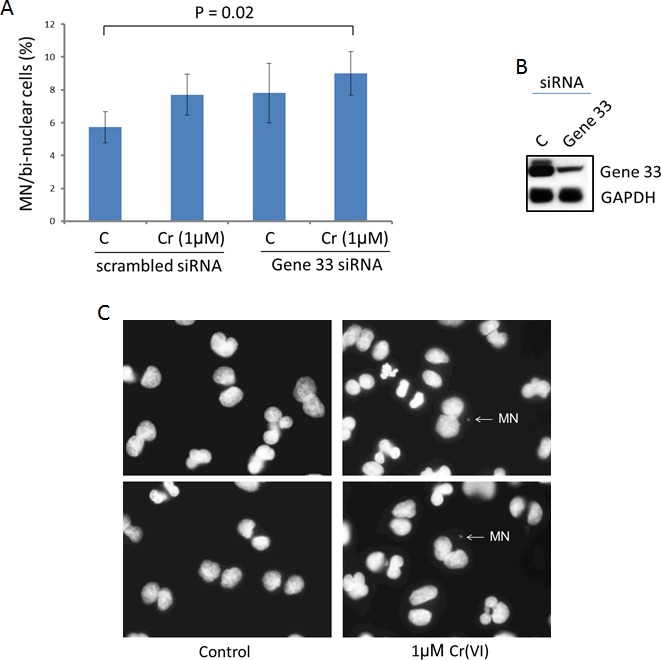
Gene 33 depletion enhances Cr(VI)-induced micronucleus formation in BEAS-2B cells **A.** Cytokinesis-block micronucleus assay were performed as described in Materials and Methods. Asterisk indicates statistically significant. Data are presented as mean +/− SD. **B.** Cells were harvested 48 hours after transfection of siRNA oligos and subjected to Western blot to check the expression of Gene 33. GAPDH was used as a loading control. **C.** Representative images showing micronuclei associated with di-nuclei.

Cr(VI) has been shown to induce chromosome instability in form of micronucleus formation in both cellular and animal models [32, 40]. Since Cr(VI) is considered a weak mutagen, induction of chromosome instability is believed to be the main mechanism leading to genomic instability and carcinogenesis [5]. Given the effect of Gene 33 on Cr(VI) induced DNA damage we wanted to examine whether Gene 33 affects Cr(VI)-induced formation of micronuclei, an indication of DNA damage-induced chromosome instability [41]. We transiently knocked down Gene 33 with siRNA in BEAS-2B cells and assessed its effect on the level of micronucleus formation induced by Cr(VI). The result shows that the combination of Gene 33 knockdown and Cr(VI) treatment led to statistically significant increase in the MN level (Figure 5A). Figure 5B and 5C show Gene 33 knockdown by siRNA and examples of MN. Our data are in line with the notion that Gene 33 regulates Cr(VI)-induced DNA damage that lead to chromosome instability in lung epithelial cells.

### Gene 33 deletion increases Cr(VI)-induced cell transformation

**Figure 6:**
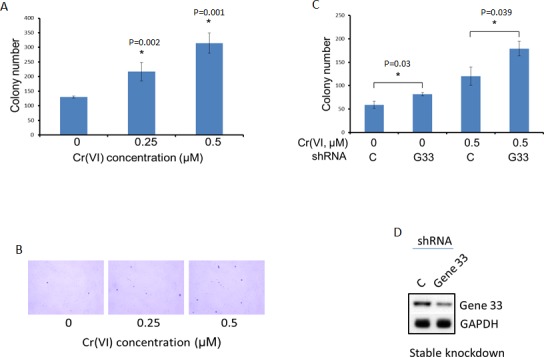
Gene 33 depletion enhances Cr(VI)-induced cell transformation in BEAS-2B cells Anchorage-independent cell growth measured by soft agar colony formation assay was used as a measure of cell transformation. **A.** BEAS-2B cells were cultured with media containing Cr(VI) of the indicated concentrations for 5 weeks before performing soft agar colony formation assay as described in Materials and Methods. **B.** Examples of the colonies from A are shown. **C.** BEAS-2B cells stably transfected with a scrambled C. or Gene 33 (G33) shRNA plasmids (Sigma) were cultured with media containing 0.5μM of Cr(VI) for 5 weeks before conducting soft agar colony formation assay as described in Materials and Methods. **D.** Western blot showing the expression of the Gene 33 in cells stably transfected with scrambled C. or Gene 33 shRNA used in in C. Data are presented as mean +/− SD (A&C).

The initial biological step of lung carcinogenesis is the malignant transformation of lung epithelial cells. Given the effect of Gene 33 on DNA damage and chromosome instability, it is likely that Gene 33 regulate Cr(VI)-induced lung epithelial cell transformation. We checked how the Gene 33 expression level affects anchorage-independent cell growth, a hallmark of cell transformation, in BEAS-2B cells using standard soft agar colony formation assay. Cr(VI) has been well documented to be capable of transforming BEAS-2B cells [33, 42, 43]. To confirm this, we treated BEAS-2B cells with Cr(VI) at 0.25 and 0.5 μM concentrations (both are sub-lethal concentrations) [33] followed by soft agar colony formation assay as described in Materials and Methods. As shown in Figure 6A, there were significant and dose-dependent increases in numbers of colonies formed after Cr(VI) treatments, confirming the previously published findings [33]. Of note, BEAS-2B cells are known to have a basal ability of forming small colonies in soft agar assay [33]. We next compared levels of Cr(VI)-induced transformation of BEAS-2B cells with or without stable Gene 33 knockdown using the same experimental protocol. We find that cells with stable Gene 33 knockdown exhibited significantly higher levels of colony formation both at the basal condition and after Cr(VI) treatments (Figure 6C). Figure 6D shows the significant lower level of Gene 33 expression in BEAS-2B cell with stable Gene 33 knockdown before transformation experiment. Our data establish that Gene 33 suppresses Cr(VI)-induced lung epithelial cell transformation, consistent with the observation that endogenous Gene 33 reduces DNA damage and inhibits chromosome instability elicited by Cr(VI).

## DISCUSSION

The cellular effects of Cr(VI) are primarily caused by the generation free radicals [7]. The relatively non-specific nature of free radicals implies that Cr(VI) likely alters multiple cellular activities, as indicated by studies on gene expression profiles [33, 44-46]. Accordingly, pathways that control cell proliferation, growth, survival, autophagy, as well as DNA damage and repair have all been shown to be part of the cellular responses to Cr(VI) [2]. Moreover, recent studies suggest that the PI3K/AKT/GSK3/β-catenin and the EGFR signaling pathways are important for Cr(VI)-induced BEAS-2B lung epithelial cell transformation [47, 48]. Given the complexity the regulation, it is crucial to identify the molecular players that drive Cr(VI)-induced lung carcinogenesis rather than those associated only with the transient stress responses.

The present study provides evidence for a novel association of the adaptor protein Gene 33 in Cr(VI)-induced lung epithelial cell transformation and reveals a previously unidentified function of this protein in the DNA damage response. We find that the Gene 33 level can be inhibited by Cr(VI) exposure in lung epithelial and lung cancer cells at the protein level and to a less extent the mRNA level. We show that Gene 33 inhibits DNA damage induced by Cr(VI). We further demonstrate that Gene 33 inhibits Cr(VI)-induced MN formation and cell transformation in lung epithelial cells.

Our data reveal that the root cause of the suppression of Gene 33 by Cr(VI) is the generation of free radicals (Figure 2A). These free radicals are apparently able to inhibit Gene 33 at both the protein and mRNA levels (Figures 1 and 2). The 26S proteasome system is clearly involved in the Cr(VI) suppression of the Gene 33 protein level (Figure 2A, 2B, 2C). However, the effect of Cr(VI) on ubiquitination of Gene 33 is interesting as Cr(VI) inhibits rather than enhance Gene 33 ubiquitination (Figure 2C). Cr(VI) also inhibits the ubiquitination of many other cellular proteins (Figure 2D). These data do not support the notion that Cr(VI) promotes Gene 33 degradation by enhancing its ubiquitination. Instead, Cr(VI) appears to suppress Gene 33 protein, at least partially, through a ubiquitin-dependent but proteasome-independent pathway or by activating the proteasome itself. Further investigation into this question is clearly needed.

The finding that inhibition of miR-200c strongly elevates the Gene 33 protein level is consistent with the existence of multiple consensus binding element for miR200 and the previous findings (Figure 2G) [35]. However, we did not detect a role of miR-200c in Cr(VI)-mediated suppression of Gene 33 despite the increase in miR-200c expression upon Cr(VI) treatment (Figure 2G and 2I).

A recent study suggests that Cr(VI)-induced DSBs accumulate at the euchromatin region where active transcription occurs [10]. These events likely slow down transcription of many genes. Whether Gene 33 is one on the genes that is suppressed by this mechanism remains to be determined. In addition, epigenetic modifications could also supress Gene 33 expression, especially in chronic setting. Further research in this direction is needed.

It has been shown that Cr(VI)-induced DSBs mainly happen at the S phase of the cell cycle as a result of the formation of Cr(III)-DNA adducts during Cr(VI) reduction process [2]. Our data confirm this notion. However, our results also seem to suggest that cells in G1 phase are also sensitive to Cr(VI)-induced DSBs. Since DNA damage induced by genotoxic agent typically happens during DNA replication in S phase [37], the apparent increase in the level of γH2AX in cells in G1 phase likely reflected DNA damage in cells entering the S phase when their DNA content was not appreciably increased comparing to G1 cells. This is similar to the cells treated with Camptothecin (Figure 3G), the drug that induces γH2AX exclusively in S phase [37]. Indeed, our experiment on the effect of Cr(VI) in A549 cells confirmed this notion (Figure S1 and the discussion therein). The high basal level of γH2AX in G2/M phase observed in our experiments was likely a result of DNA damage-independent mitotic phosphorylation of H2AX [49, 50]. Consistently, the γH2AX level was less affected by Cr(VI) during G2/M phase (Figure 3D).

We find that P-p53 behaved differently between 2 hour and 6 hour treatments with Cr(VI): a dose-dependent reduction vs. a dose-dependent increase except at 20 μM (Figure 3B and 3C). These data, coupled with those showing limited increase in the level of γH2AX in response to Cr(VI) at 2 hours vs. strong increase in the level of γH2AX at 6 hours, suggest that the kinase that was responsible for the basal phosphorylation of p53 was inhibited by Cr(VI) at the initial period of the treatment before DNA damage occurs. Our results show that the activities of p38 and ERK MAPK kinases, indicated by the levels of p38 and ERK phosphorylation, were mostly unchanged by 6 hour Cr(VI) treatment (Figure 4A). Although these two kinases have been shown to phosphorylate p53 at Serine 15 [51], our data do not support roles of these kinases in phosphorylation of p53 at serine 15 in response to Cr(VI). Of note, it has been reported that ERK phosphorylates p53 at serine 15 in response to Cr(VI) in A549 cells, albeit at a much higher concentration (50μM)[39]. Moreover, we were unable to detect any signal of P-p53 in A549 cells after 24 hour treatment with 5 μM Cr(VI) (Figure 4D). Thus, a cell type- or dose-dependent discrepancy likely exists.

Our observation that depletion of Gene 33 protein significantly elevates Cr(VI)-induced DSBs (Figure 4) suggests Gene 33 may either function to prevent Cr(VI)-induced DNA damage or to facilitate DNA repair following the DNA damage. We also observed that Gene 33 depletion led to dramatically elevated levels of ubiquitinated γH2AX upon Cr(VI) treatment (Figure 4A and 4B), consistent with the notion that ubiquitinated H2AX is associated with the DNA damage response [52]. Our data showing that Gene 33 can suppress Cr(VI)-induced micronucleus formation and cell transformation is consistent with the notion that Gene 33 functions to inhibit Cr(VI)-mediated DNA damage and chromosome instability.

The mechanism underlying the regulation of Cr(VI)-induced DNA damage by Gene 33 is unclear. It has been shown that EGFR translocates to the nucleus after certain genotoxic insults and facilitates DNA repair by activating DNA-PK and phosphorylating histone H4 [53-55]. As Gene 33 is a well-established regulator of the EGFR kinase activity and its internalization [21, 22, 56, 57], it is tempting to speculate that Gene 33 may modulate EGFR nuclear translocation to and/or activity in the nucleus thereby regulating the DNA repair process. Our experiments indeed show that Gene 33 is capable of affecting the interaction of EGFR with DNA-PK and histone H4 (SP and DX, unpublished observations). Further in-depth investigation into this mechanism is currently underway.

The effect of Gene 33 on canonical EGFR signaling pathway may also need to be taken into consideration when evaluating its role in Cr(VI) carcinogenesis. Our data show that Gene 33 knockdown had limited effect on cell cycle progression in BEAS-2B cells cultured in medium containing 10% FBS (Figure 4E and 4F). Since Gene 33 is a specific inhibitor of EGFR family receptor tyrosine kinases [20-22, 58], it is conceivable that while Gene 33 can significantly inhibit EGF-induced cell cycle entry under serum deprivation [20], its role in cell cycle progression in presence of 10% FBS may be limited. However, long term increase in EGFR signaling likely contributes to higher potential for cell transformation. Accordingly, elevated activity of the EGFR signaling pathway as a result of increased expression of EGFR has been reported in Cr(VI)-transformed BEAS-2B cells [48]. The inhibition of Gene 33 expression by Cr(VI) would be consistent with the increased EGFR expression and EGFR signaling as Gene 33 inhibits EGFR kinase activity and promotes EGFR degradation [57, 58]. It will be interesting to determine whether the reduced expression of Gene 33 contributes to the elevated EGFR signaling in Cr(VI)-transformed lung epithelial cells. Furthermore, the involvement of other known functions of Gene 33 in cell apoptosis, cell senescence, and cell migration in Cr(VI)-mediated cell transformation and tumorigenesis also warrants further investigation.

**Figure 7:**
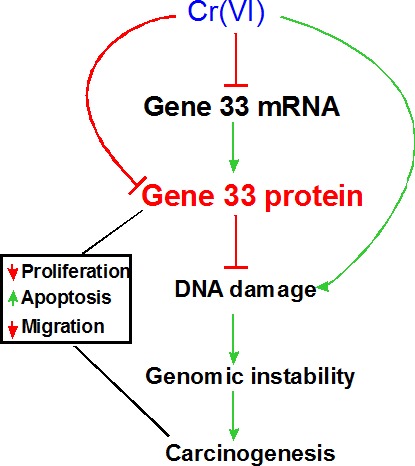
Proposed model for Gene 33 in Cr(VI)-induced lung carcinogenesis Cr(VI) reduces the levels of Gene 33 mRNA and protein thereby inhibiting Cr(VI)-induced DNA damage that lead to genomic instability, cell transformation and carcinogenesis. Gene 33 may also reduce lung carcinogenesis by inhibiting lung epithelial cell proliferation, promoting apoptosis, and slowing down cell migration.

Taken together, the present study implicates Gene 33 as an important player in Cr(VI)-induced lung epithelial transformation and lung carcinogenesis. Although these findings are confirmative of the previous view that Gene 33 is a tumor suppressor protein, this is the first study linking Gene 33 to potential lung malignancy induced by an environmental and occupational carcinogen. In addition, we explored the mechanism underlying the suppression of Gene 33 expression by Cr(VI). Moreover, we have identified a novel association of Gene 33 with Cr(VI)-induced DNA damage. We propose a model on the potential role of Gene 33 in Cr(VI)-induced lung carcinogenesis: 1. Cr(VI) inhibits Gene 33 expression at both mRNA and protein levels. 2. Gene 33 inhibits Cr(VI)-induced DNA damage, genomic instability, cell neoplastic transformation, and likely carcinogenesis. 3. This mechanism, together with other functions of Gene 33 in cell proliferation, apoptosis, and cell migration, constitutes the overall role of Gene 33 in Cr(VI)-induced lung carcinogenesis (Figure 7).

## MATERIALS AND METHODS

### Cell lines, antibodies, and plasmids

BEAS-2B human lung bronchial epithelial cells and A549 human lung adenocarcinoma cells were obtained from American Type Culture Collection. Cells were cultured in DMEM supplemented with 10% fetal bovine serum (FBS) and antibiotics (100 unit/ml of penicillin and 100 μg/ml of streptomycin sulfate per mL) under 5% CO_2_ condition. The antibody to Gene 33 has been described previously [20, 24]. The antibody to γH2AX was purchased from Biolegend. Antibodies to GAPDH, EGFR, and β-actin were purchased from Santa Cruz Biotechnology. The antibody to P-p53 (S15) was purchased from Cell Signaling Technology. The mammalian expression plasmid encoding FLAG-tagged Gene 33 have been described previously [20]. The mammalian expression plasmid encoding FLAG-tagged ubiquitin was a generous gift from Dr. Wei Dai at New York University School of Medicine.

### Western bolt

Standard Western blot procedure was using throughout the study. SDS-PAGE was carried out using the mini gel system from Bio-Rad. Proteins in gels were then transferred to PVDF membrane. After blocking with TBST containing 5% non-fat dry milk, the membrane was incubated overnight with primary antibodies diluted with TBST containing 5% BSA using dilutions suggested by the manufacturers. After thorough wash with TBST, the membrane was further incubated with horse radish peroxidase-conjugated secondary antibodies for 1 hour at room temperature followed by thorough washing with TBST buffer. The signals on the membrane were developed with an ECL system (Pierce).

### Immunoprecipitation

Cells were lysed in the RIPA buffer supplemented with protease and phosphatase inhibitors. The lysates were then centrifuged for 30 min at >10000g. The supernatants were kept as total cell lysates. One μg of the antibody and 40 μl of protein G Agarose resin (50/50, Upstate Biotechnology) were then added to 1 mg of each cell lysates and incubated at 4°C for overnight followed by extensively washing with the lysis buffer. Proteins bond to the resin were then extracted with SDS sample buffer and subjected to SDS-PAGE followed by Western blot with appropriate antibodies.

### Laser scanning cytometry

Cells were cultured on 2-well chamber slides. After treatments, cells were fixed with 1% formaldehyde followed by 70% ethanol, pemmeabilized with 0.1% triton X100, blocked with 1% BSA, and immunostained with primary antibodies to both γH2AX and phospho-p53 (S15) followed by fluorescent-conjugated secondary antibodies together with DAPI. The cells were then analyzed with CompuCyte four laser iCys scanning cytometer.

### Real-time PCR

Cells were treated and harvest for total RNAs with Trizol reagent (Life technologies). Total RNAs were converted to cDNA using a RT-PCR kit (Clontech) and subjected to real-time PCR using SYBR Green master mix (Clontech) and a real-time PCR machine (StratageneMx3005P). The primers used are follows: Gene 33: 5′-CTGGAGCAGTCGCAGTGAG-3′/ 5′-GCCATTCATCGGAGCAGATTTG-3′, GAPDH (as internal control): 5′-ACAACTTTGGTATCGTGGAAGG-3′/ 5′-GCCATCACGCCACAGTTTC-3′ real-time PCRs for microRNAs were carried out using miScript Primer Assay (Qiagen) according to manufacturer's instructions. The results were corrected for U6.

### RNAi and microRNA

For transient RNAi, cells were cultured to 70% confluence and transfected with scrambled or Gene 33 siRNA oligos (Dharmacon) using Lipofectamine 2000 according to manufacturer provided protocol. Cells were analyzed at least 48h post-transfection. For microRNA inhibition, a negative control or a miR-200c microRNA inhibitor (Dharmacon) was transfected (at 25 nM) using Lipofectamine RNAiMAX reagent (Life technology) and analyzed 24 hours after transfection. For stable knockdown, shRNA constructs containing scrambled or Gene 33 (Sigma-Aldrich) were transfected into BEAS-2B cells using Lipofectamine 2000 (Life technology) followed by selection with puromycin.

### Cytokinesis-block micronucleus assay

BEAS-2B cells growing on a 6 well plate were transfected with scrambled or Gene 33 siRNA oligos. Forty eight hours after transfection, cells were passed to 2-well chamber slides. Twenty four hours later, cells were treated with cytochalasin B (9μg/ml) for additional 24h with or without co-treatment with1μM Na_2_CrO_4_. At the end of the treatments, cells were fixed in 1% formaldehyde and stained with DAPI (2.85μg/ml). Numbers of micronuclei in 1000 binucleated cells were scored under a fluorescence microscope and the ratio of micronuclei and binucleated cells were presented as percentage.

### Soft agar colony formation assay

Cells were plated in 0.3% top agar medium on 0.5 % bottom agar medium in 6-well plates (5,000 cells/well) in triplicates. The plates were incubated at 37°C for 4 weeks. At the end of the incubation, plates were staining with Crystal Violet and air-dry. Total numbers of colonies in each well were then counted.

## SUPPLEMENTARY MATERIAL FIGURE


