# Supplementary material for: Gene 33/Mig6 inhibits hexavalent chromium-induced DNA damage and cell transformation in human lung epithelial cells
Source: Oncotarget. 2016 Jan 9;7(8):8916–30. doi: 10.18632/oncotarget.6866 (PMC4891014; doi:10.18632/oncotarget.6866)
Supplement: Supplementary file 1 [file oncotarget-07-8916-s001.pdf]

Gene 33/Mig6 inhibits hexavalent chromium-induced DNA damage and cell transformation in human lung epithelial cells

Supplementary Material

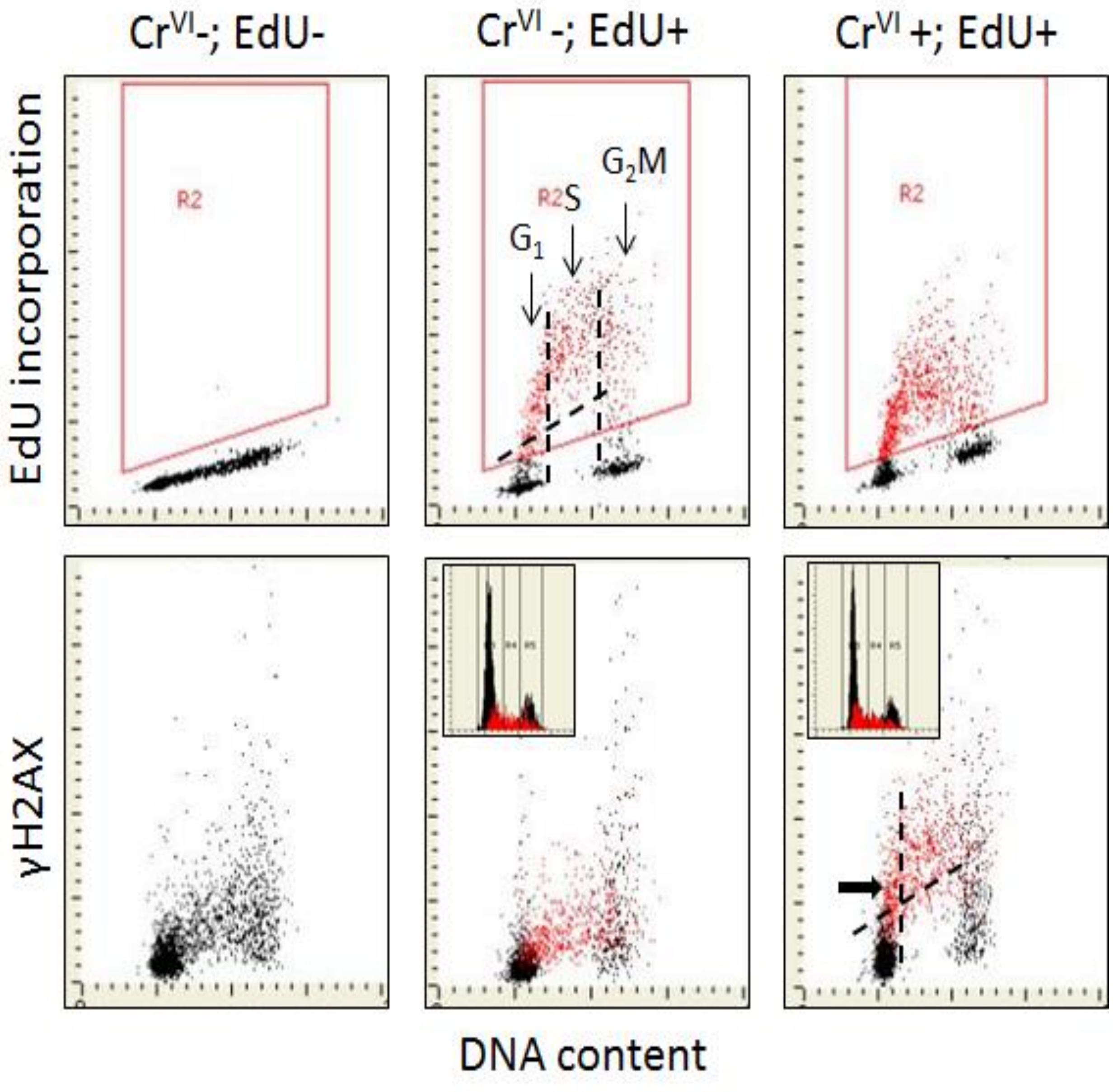

Figure S1. Cr(VI) induces maximal and immediate DNA damage to DNA replicating cells.

Fig. 3 appears to show that Cr(VI) induces significant DNA damage, measured as H2AX phosphorylation, not only to cells in S phase but also at the G1 phase of the cell cycle. To test

whether these are early S phase cells that just started DNA replication and therefore have DNA content very close to that of G1 cells we carried on experiments to correlate the Cr(VI) induced H2AX phosphorylation with actual DNA replication. A549 cells were untreated or treated in culture with 5 μM Cr(VI) for 2h. At the end of Cr(VI) treatment cells with were labeled with 20 μM of the DNA precursor 5-ethynyl-2'-deoxyuridine (EdU) for 1 h. All cells were then counterstained with DAPI, the stoichiometric marker of DNA content which reveals the cell cycle phase. Cells' red fluorescence representing EdU incorporation and green denoting γH2AX expression have been measured by LSC using gating analysis as shown in Fig. 3. The cells incorporating EdU were gated and electronically colored red. The DNA content histograms from the respective cultures are shown in inserts of the panels (Suppl. Fig. 1).

The data reveal that cells exposure to Cr(VI) for 2 h decreased amount of incorporated EdU, an indication of a suppression of DNA replication rate. It also increased cells heterogeneity in terms of a degree of EdU incorporation (compare Cr(VI)-;EdU+ with Cr(VI)+,EdU+. These cells showed also evidence of extensive phosphorylation of H2AX. Note that in the Cr(VI)-treated culture most S phase cells show distinctly elevated H2AX expression), above the control threshold (marked as a skewed dashed line), which represents the level of constitutive DNA damage by endogenous oxidants. Of importance is the observation that essentially all cells from the Cr(VI) treated culture with the level of γH2AX above the control are the cells replicating DNA. Thus, even the cells with DNA content close to that of G1 cells (marked by thick horizontal arrow) were incorporating EdU. These data indicate that those apparent G1 cells containing DNA damage in response to Cr(VI) (Fig. 3) were in fact early S phase cells just starting DNA replication and thus with the DNA content very close to that of G1. This confirms the notion that Cr(VI)-induced DNA damage occurs almost exclusively in the DNA replicating cells.
